# Exosomal cell-to-cell transmission of alpha synuclein oligomers

**DOI:** 10.1186/1750-1326-7-42

**Published:** 2012-08-24

**Authors:** Karin M Danzer, Lisa R Kranich, Wolfgang P Ruf, Ozge Cagsal-Getkin, Ashley R Winslow, Liya Zhu, Charles R Vanderburg, Pamela J McLean

**Affiliations:** 1 MassGeneral Institute for Neurodegenerative Disease, Department of Neurology, Massachusetts General Hospital, Charlestown, MA, USA; 2 Advanced Tissue Resource Center, Harvard NeuroDiscovery Center, Harvard Medical School, Boston, MA, USA; 3 Present address: Neurology Department, University of Ulm, Ulm, Germany; 4 Present address: Department of Neuroscience, Mayo Clinic, 4500 San Pablo Rd, Jacksonville, FL, 32224, USA

**Keywords:** Alpha synuclein, Oligomers, Exosomes, Parkinson’s disease, Aggregation, Secretion

## Abstract

**Background:**

Aggregation of alpha-synuclein (αsyn) and resulting cytotoxicity is a hallmark of sporadic and familial Parkinson’s disease (PD) as well as dementia with Lewy bodies, with recent evidence implicating oligomeric and pre-fibrillar forms of αsyn as the pathogenic species. Recent *in vitro* studies support the idea of transcellular spread of extracellular, secreted αsyn across membranes. The aim of this study is to characterize the transcellular spread of αsyn oligomers and determine their extracellular location.

**Results:**

Using a novel protein fragment complementation assay where αsyn is fused to non-bioluminescent amino-or carboxy-terminus fragments of humanized Gaussia Luciferase we demonstrate here that αsyn oligomers can be found in at least two extracellular fractions: either associated with exosomes or free. Exosome-associated αsyn oligomers are more likely to be taken up by recipient cells and can induce more toxicity compared to *free* αsyn oligomers. Specifically, we determine that αsyn oligomers are present on both the outside as well as inside of exosomes. Notably, the pathway of secretion of αsyn oligomers is strongly influenced by autophagic activity.

**Conclusions:**

Our data suggest that αsyn may be secreted via different secretory pathways. We hypothesize that exosome-mediated release of αsyn oligomers is a mechanism whereby cells clear toxic αsyn oligomers when autophagic mechanisms fail to be sufficient. Preventing the early events in αsyn exosomal release and uptake by inducing autophagy may be a novel approach to halt disease spreading in PD and other synucleinopathies.

## Background

Parkinson’s disease (PD) is pathologically characterized by alpha-synuclein (αsyn) immunopositive intracellular deposits termed Lewy bodies [1]. Gene multiplication of the αsyn gene [2,3] and missense mutations [4-6] are linked to familial forms of PD. Together, these data support a role for αsyn in the pathogenesis of PD.

Because αsyn inclusion body pathology associated with PD occurs in a hierarchical distribution with its epicenter in the brainstem, then extends to the mesolimbic cortex and associated areas [7], Braak et al. have suggested that αsyn pathology spreads gradually throughout the neuraxis as PD progresses [8]. However, as yet, the underlying mechanisms of disease progression in PD remain to be determined.

The main component of Lewy bodies and Lewy neurites are fibrillar aggregates of αsyn [1] but a growing body of evidence suggests that prefibrillar oligomers of αsyn are key contributors in the progression of Parkinson's disease [9-15].

Until recently αsyn was thought to exert its toxic effects intracellularly. However, this concept was challenged when El-Agnaf et al. detected αsyn species in human plasma and CSF [12]. Furthermore, Desplats et al. demonstrated that αsyn can be directly transmitted from neuronal cells overexpressing αsyn to transplanted embryonic stem cells both in tissue culture and in transgenic animals [16]. Concurrently, our group was able to demonstrate that cell produced αsyn oligomers are secreted and taken up by neighboring cells where they have detrimental consequences [17]. These results suggest that the pathogenic action of αsyn oligomers are not limited to the donor cells but can extend into the extracellular space and affect neighboring cells. In support of this hypothesis, recombinant αsyn oligomers added to cell culture medium have been shown to be internalized by recipient cells causing either cell death or seeding of αsyn [18-22]. The mechanism(s) of αsyn transmission from cell to cell that contribute to the spread of αsyn pathology remain largely unknown. One intriguing question is how intracellularly generated αsyn is released into the extracellular space. A first hint that αsyn may be secreted by externalized vesicles that have hallmarks of exosomes was recently provided [23,24].

The aim of this current study is to characterize αsyn associated with exosomes and to explore the nature of αsyn secretion using a highly sensitive protein complementration assay [25-30]. Moreover, we examine the specific relationship of αsyn oligomers with exosomes and find that both intra-and extra-exosomal associated αsyn oligomers exist. The importance of intact exosomes for re-uptake of αsyn oligomers into neighboring cells and the role of autophagic activity on exosomal secretion of αsyn oligomers are also examined.

## Results

### Alpha-synuclein oligomers are found in exosomes

Increasing evidence suggests that αsyn can be released by neurons and neuronal like cells [17,31-33] although extracellular αsyn and its pathological relevance are still hotly debated in the field. Recent work from our own group and the elegant study from Desplats et al. suggest that αsyn can be transferred from cell to cell and thus may provide an explanation for the spread of αsyn pathology in PD patients [16,17]. However, little is known about the mechanism of αsyn secretion.

Recently, secretion of αsyn in association with membrane vesicles, identified as exosomes based on their composition and biophysical properties, has been described [23,24]. However, the specific αsyn species (monomers vs pathogenic oligomers) secreted with exosomes and the location of αsyn remains to be determined.

To investigate whether oligomeric species of αsyn are present in the exosome enriched fractions we employed a bioluminescent protein-fragment complementation assay [25,26,30]. In this strategy, αsyn was fused to non-bioluminescent amino-terminal (S1) or carboxy-terminal fragments (S2) of *Gaussia princeps* luciferase [28] that can reconstitute when brought together by αsyn-αsyn interactions [25], thus providing a readout of αsyn oligomerization (Additional file 1: Figure S1A). The same principle of protein complementation with fluorescent venus YFP was used generating the fluorescent protein-fragment complementation pair V1S or SV2 whereby N-terminal half of Venus YFP is fused to the N-terminus of αsyn (V1S) and C-terminal half of Venus YFP is fused to the C-terminus of αsyn (SV2) [25] (Additional file 1: Figure S1B).

**Figure 1:**
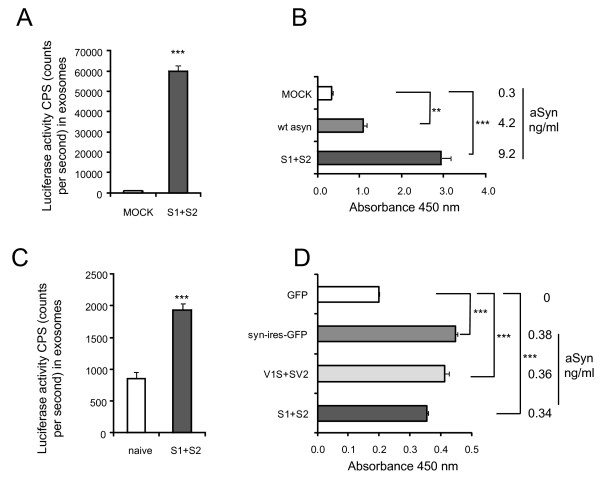
**Extracellular αSyn oligomers are associated with exosomes: (A) Exosomal fractions from human H4 cells transfected with αsyn complementation pair S1 and S2 contain high amounts of αsyn oligomers, analyzed with a luciferase assay.** Signal corresponds to 1.25x100mm dishes. n = 6, unpaired t test with Welch's correction, ***p < 0.001 (**B**) αsyn ELISA analysis from exosomal fractions from human H4 cells transfected with S1/S2, wt αsyn or Mock. Signal corresponds to 1x100 mm dish. n = 4, unpaired t test with Welch's correction, ***p < 0.001 (**C**) Exosomal fractions from primary neurons infected with αsyn complementation pair S1 and S2 contain high amounts of αsyn oligomers, analyzed with a luciferase assay, signal corresponds to 1x60 mm dish, n = 4, unpaired t test with Welch's correction, ***p < 0.001 (**D**) αsyn ELISA analysis from exosomal fractions from primary neurons infected with AAV expressing GFP, αsyn -ires-GFP, V1S/SV2 and S1/S2. Values correspond to 1x60 mm dish, n = 4, Tukey's Multiple Comparison Test, ***p < 0.001.

Human H4 neuroglioma cells were co-transfected with S1 and S2 that reconstitute luciferase activity upon αsyn oligomerization. Exosomes were isolated from conditioned media (CM) of H4 cells using an established subcellular fractionation methodology [34,35] and the exosomal pellet was analyzed for luciferase activity that is indicative of αsyn oligomers. Interestingly, we witnessed a large increase in luciferase activity in the exosomal fraction derived from H4 cells transfected with S1 and S2 compared to exosomes from mock transfected cells (Figure 1A), suggesting that αsyn, and specifically αsyn *oligomers* are present in the exosomal fraction. To exclude the possibility that N- or C-terminal fragments of human Gaussia Luciferase can interfere with protein sorting in exosomes, our results were verified in exosomes isolated from human H4 cells transfected with untagged wild-type (wt) αsyn using a human αsyn ELISA. Significant levels of αsyn were present in the exosomal fraction from wt αsyn and S1/S2 transfected H4 cells compared to exosomes derived from empty vector (mock) transfected cells (Figure 1B).

We extended these observations to primary cortical neurons where αsyn oligomers were also found in the exosomal fraction isolated from primary neurons co-transduced with adeno-associated virus (AAV) encoding S1 (AAV-S1) and S2 (AAV-S2) as demonstrated by a significant increase in luciferase activity compared to exosomes isolated from naive neurons (Figure 1C). In accord with the experiments performed in H4 cells, we also confirmed the presence of αsyn in exosomes derived from primary neurons infected with a variety of different AAV constructs encoding either αsyn-ires-GFP, AAV-S1 and AAV-S2 or αsyn-venusYFP fluorescent protein-fragment complementation pair (AAV8-V1S or AAV8-SV2) (Figure 1D) using an αsyn ELISA. Taken together, our data provide evidence that αsyn *oligomers* are present in the exosomal fractions from both neurons and non-neuronal cells.

### Characterization of exosomes

**Figure 2:**
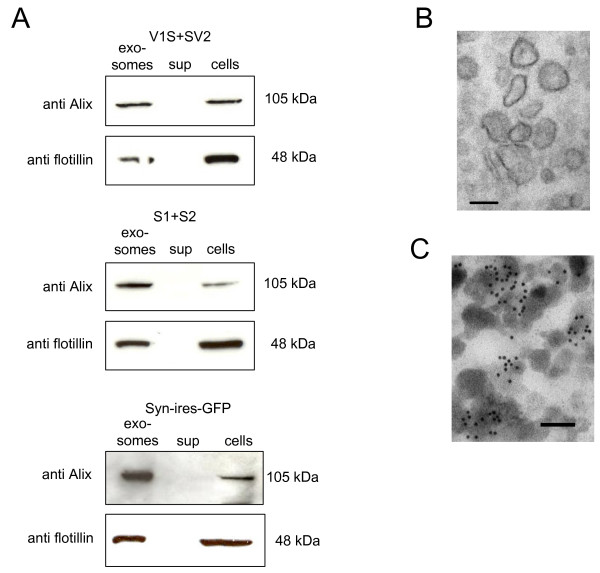
**Characterization of exosomes: (A) Exosomal pellets derived from primary neurons infected with AAV-V1S/SV2, AAV-S1/S2, AAV- αsyn-ires-GFP were resuspended in 1xPBS and analyzed by Western blotting using exosome specific antibodies anti Alix and anti flotillin.** Also total cell lysates and exosome free supernatant (sup) were loaded as positive and negative controls, respectively. (**B**) Exosomes from S1/S2 transfected H4 cells were prepared as described before. After fixation vesicles were negatively stained with 2% uranyl acetate and observed under electron microscopy. Scale bar, 100 nm. (**C**) Exosomes from primary neurons infected with AAV-V1S/SV2 were negatively stained with 2% uranyl acetate and immunolabeled with CD63 antibody as exosomal marker. Scale bar, 100 nm.

To confirm the presence of exosomes, fractions from both primary neurons and H4 cells were subjected to SDS-PAGE and immunoblotting. All exosomal fractions were found to be immunopositive for the exosome-specific proteins alix and flotillin, whereas the ‘exosome-free’ supernatant was immuno-negative for alix and flotillin (Figure 2A).

Moreover, exosome-enriched fractions isolated from CM of H4 cells transfected with the αsyn complementation pair S1 and S2 were also analyzed using electron microscopy and demonstrated the distinctive vesicular morphological structures characteristic of exosomes (Figure 2B). Immuno-electron microscopy with an antibody against the exosomal marker CD63 [36], confirmed characteristic exosomal vesicles typically 60–100 nm in size in exosome enriched fractions from CM of primary neurons co-transduced with AAV expressing the αsyn complementation pair V1S or SV2 (Figure 2C).

Because microRNAs (miRs) have been found in exosomes [37], miR profiling is a powerful tool to definitively characterize exosomes. Exosome fractions from both S1/S2 transfected H4 cells and primary neurons transduced with AAV- αsyn-ires-GFP were found to contain a large number of miRs that have previously been reported to be present in exosomes (see Tables 1 and 2). Of interest, we did not detect miR-7, which has been previously identified as a negative regulator of αsyn expression [66].

**Table 1:** MicroRNA profiling of alpha-synuclein containing exosomes from H4 cell culture media

**miRNAs**	**function**	**remarks**	**Key citations**
7b	Inhibition of cell cycle progression and growth of melanoma cells	present in exosomes	[38,39]
7i	regulatory responses to microbial infection	present in exosomes	[40,41]
18b	exocytosis, angiogenesis, hematopoiesis, tumorgenesis	present in exosomes	[40]
182	Repression of tumor suppressors	present in exosomes	[39,42]
184	Block of NFAT1 protein expression	present in exosomes	[40,43]
363*	-		[44,45]
373*	Involvement in tumor migration and invasion	present in exosomes	[39,46]
375	exocytosis, angiogenesis, hematopoiesis, tumorgenesis	present in exosomes	[39,40]
376*	-		[47,48]
424	controlling the macrophage differentiation program	present in exosomes	[39,49]
431	-	CNS specific miRNA	[47,50]
455	-		[51]
487a	Role in angiogenesis and cell aging		[52]
492	-		[44]
494	regulation of PTEN expression and functions as a micro-oncogene in carcinogenesis	present in exosomes	[39,53]
518c	-		[44,54]

**Table 2:** MicroRNA profiling of alpha-synuclein containing exosomes from primary cortical neuron media

**miRNAs**	**function**	**remarks**	**Key citations**
27a	activating the expression of P-glycoprotein	present in exosomes	[39][55]
28	inhibitor of thrombopoietin receptor translation	present in exosomes	[39][56]
34a	suppression of cell proliferation through modulation of the E2F signaling pathway	-	[57]
106b	modulation of TGFβ signaling in tumors	present in exosomes	[39][58]
184	Block of NFAT1 protein expression	present in exosomes	[40,43]
185	Inducion of cell cycle arrest in lung cancer cell lines	present in exosomes	[40,59]
192	affects cellular proliferation through the p53-miRNA circuit	present in exosomes	[39][60]
199b	Regulation of protein phosphatase 2A inhibitor) in human choriocarcinoma	-	[61]
302b*	regulatory mechanism in tuning stem cell properties	-	[62]
373*	Involvement in tumor migration and invasion	present in exosomes	[39,46]
422b	-	present in exosomes	[40]
431	-	CNS specific miRNA	[47,50]
448	-	-	[63]
455	-	-	[51]
487a	Role in angiogenesis and cell aging	-	[52]
491	decreases cell viability by induction of apoptosis	-	[64]
493-3p	-	-	[54]
518*	Predicted to be involved in Huntington’s disease	-	[54,65]
522	-	present in exosomes	[39]
526a	-	present in exosomes	[39]
526b	-		

### Localization of αsyn oligomers in the extracellular space

**Figure 3:**
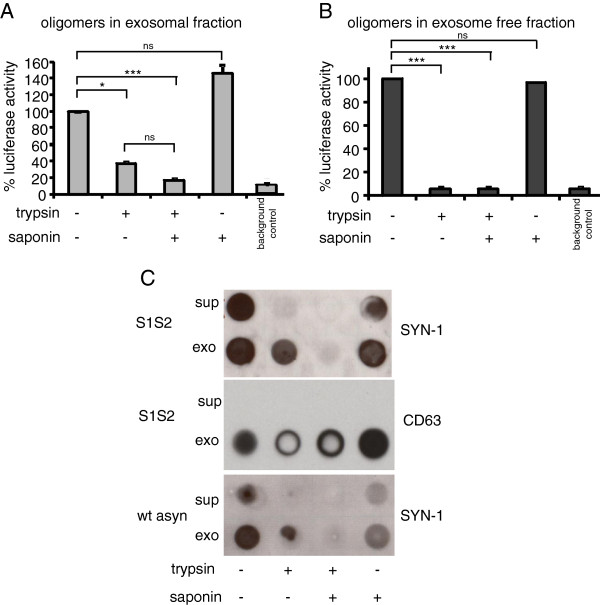
**Localization of αsyn oligomers within exosomes: (A) **Luciferase assay with exosomes derived from human H4 cells transfected with S1/S2 or 0.1% saponin/or 0.1% saponin. (**B**) Luciferase assay with exosome-free supernatants after αsyn oligomer digestion using 0.25% trysin and/or 0.1% saponin. (**C**) Dot blot approach with exosomal fraction (exo) and exosome free supernatant (sup) from H4 cells transfected with S1/S2 after αsyn oligomer digestion using 0.25% trysin and/or 0.1% saponin. Probing with syn-1 antibody shows only a complete digestion of αsyn oligomers when trypsin and saponin are used concurrently. Signal of exosomal marker CD63 is abolished by addition of solely trypsin.

Cytosolic proteins can be secreted from cells via at least two distinct pathways which include exocytosis and fusion of multi-vesicular bodies with the plasma membrane to release exosomes. Defining the localization of αsyn in the extracellular space will provide insight into the mechanisms and pathways involved in αsyn release. To examine the localization of αsyn oligomers in the extracellular space we first digested exosome-enriched fractions containing αsyn S1/S2 oligomers with 0.25% trypsin. Interestingly, trypsin digestion significantly reduced luciferase activity in the exosome fraction by 62% (Figure 3A) suggesting the presence of αsyn oligomers either on the external surface of the exosomes or outside of exosomes. However, trypsin treatment did not eliminate luciferase activity to background levels completely, indicating that αsyn oligomers must exist in the lumen of the exosomes that are insensitive to the trypsin treatment. To further clarify, we cotreated exosome fractions with trypsin and the detergent saponin (0.1%). The presence of saponin results in exosome-membrane permeabilization. Notably, we saw a complete elimination of luciferase activity in the presence of trypsin and saponin (Figure 3A). Treatment with saponin alone slightly increased luciferase activity compared to untreated control exosomes, although it was not a significant increase. This could be due to enhanced substrate availability to lumenal αsyn oligomers. The same experimental paradigm was tested on the "exosome-free" supernatant fraction. As expected, trypsin eliminated all luciferase activity from "free" αsyn oligomers in the supernatant fraction in the presence or absence of saponin (Figure 3B). These data confirm the absence of exosomes from the supernatant fraction and verify that the experimental paradigm is sufficient to digest all available αsyn oligomers.

To confirm our results on the localization of αsyn oligomers inside/outside exosomes we examined samples prepared under the same experimental conditions using dot blot immunoblotting. Probing with Syn-1 antibody showed that exsosome free αsyn oligomers (sup) were completely digested by trypsin independent of saponin treatment (lane 1, sup, Figure 3C). In contrast, Syn-1 signal was not completely eliminated when exosome fractions were treated with trypsin (lane 2, exo, Figure 3C). Only the combination of trypsin and saponin resulted in a complete digestion of αsyn oligomers and a consequent abolishment of αsyn immunostaining in exosome fractions. Probing with an antibody against the exosomal marker CD63, which is known to be located solely on the outside of exosomes, shows reactivity only in the exosome fractions not treated with trypsin and no reactivity at all in supernatant-associated αsyn oligomers. Dot blots were also performed on fractions prepared from CM of cells transfected with wt untagged αsyn. As expected, trypsin treatment resulted in a reduction in Syn-1 signal in exosome associated αsyn oligomers but only the combination of trypsin and saponin resulted in a complete digestion and abolishment of Syn-1 signal. Together, the data indicate that αsyn oligomers are located on the inside and outside of exosomes.

### Exosome-associated αsyn oligomers are more prone to internalization than exosome-free αsyn oligomers

**Figure 4:**
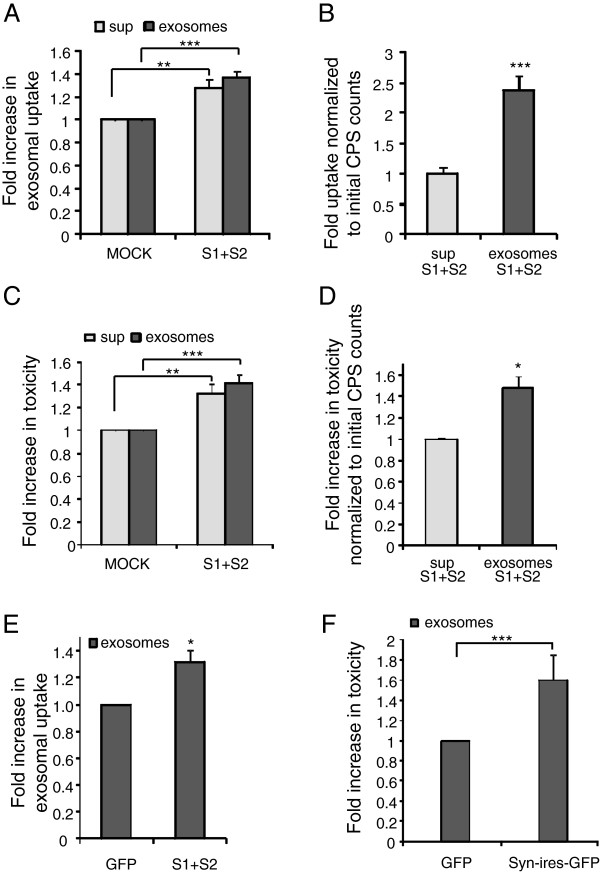
**Exosome-associated αsyn oligomers are preferentially internalized and more toxic than exosome-free αsyn oligomers.** (**A**) Exosomal fractions and exosome-free supernatants (sup) from human H4 cells transfected with S1/S2 or mock were applied to naive H4 cells and incubated for 3 days. Uptake of αsyn oligomers into recipient cells was measured by luciferase assay. n = 9, unpaired t test with Welch's correction, ***p < 0.001. (**B**) Naïve H4 cells treated with exosome-associated S1/S2 oligomers and exosome-free S1/S2 oligomers (sup) were assayed for luciferase activity. Luciferase signal was normalized to the amount of αsyn oligomers in the input, n = 9, unpaired t test with Welch's correction, ***p < 0.001. (**C**) H4 cells treated with exosome-associated αsyn fractions conferred greater toxicity on naive H4 cells than exosome-free αsyn (sup n = 5, unpaired t test with Welch's correction, ***p < 0.001 (**D**) Naïve H4 cells treated with exosome-associated αsyn oligomers and exosome free αsyn oligomers (sup) were assayed for toxicity. Level of toxicity was normalized to the luciferase activity in each input n = 5, Mann Whitney test, *p < 0.05. (**E**) Exosomal fractions derived from primary neurons infected with AAV-S1/S2 or AAV-GFP were applied to naive neurons and incubated for 3 days. Uptake of αsyn oligomers into recipient neurons was measured performing a luciferase assay on recipient cells. n = 3, one-sample t-test, *p < 0.05. (**F**) Toxicity assay of naïve primary neurons treated with exosome-enriched fractions and exosome-free fractions (sup) derived from either AAV-αsyn-ires-GFP or AAV-GFP infected primary neurons. n = 9, one sample t-test, ***p < 0.001.

It has been reported that recombinant αsyn or αsyn oligomers can be internalized by cells and result in various cellular effects [18-20,33,67]. Furthermore, we and others have shown that cell produced αsyn oligomers can be secreted and taken up by proliferating cells and primary neurons [17,23]. To investigate if exosomes are required for the internalization of αsyn oligomers, we exposed naive H4 cells to exosome-associated αsyn oligomers or exosome-free supernatant containing αsyn oligomers derived from S1/S2 transfected H4 cells for 3 days. Concurrently, exosomes or exosome-free supernatant from ”mock” transfected cells were added to naïve H4 cells. Interestingly, we found that exosome associated αsyn oligomers are more prone to being taken up than exosome free asyn oligomers (Figure 4A). To control for the variable amounts of αsyn in each exosome or supernatant preparation added to cells, the luciferase signal detected in the recipient cells was normalized back to the initial luciferase counts added to the naïve cells. Data analysed in this way revealed a 2.4 fold increase in uptake of exosome-associated αsyn oligomers compared to exosome-free αsyn oligomers (Figure 4B).

Recombinant oligomers as well as physiologically secreted αsyn oligomers can cause cell death when applied to culture medium of different cell lines and primary neurons [18-20,23,33,67]. To determine if exosome-associated αsyn oligomers confer more cytotoxicity compared to exosome-free αsyn oligomers, we applied exosome-enriched fractions or exosome-free fractions derived from S1/S2 or MOCK transfected H4 cells to naïve proliferating H4 cells and found an increase in Caspase 3/7 activation conferred by exosome associated αsyn oligomers (Figure 4C). To ensure the same amount of αsyn oligomers in each fraction, the level of Caspase 3/7 activation was normalized to the amount of αsyn oligomers prior to the addition to naïve cells. Interestingly, a significant 1.5-fold increase in Caspase3/7 activation and resulting apotosis induction from exosome-associated αsyn oligomers compared to exosome-free αsyn oligomers was detected (Figure 4D).

In accordance with our data for human H4 cells we confirmed that exosome-associated αsyn oligomers could also be taken-up by naive primary neurons (Figure 4E) and induce apoptosis as characterized by an increase in caspase3/7 activity (Figure 4F). Unfortunately, due to high levels of non-specific background bioluminescence from B-27 supplement in our neuronal cell culture medium, we were unable to assess the internalization of exosome-free αsyn oligomers by primary neurons.

### Exosomes need to be intact to be internalized

**Figure 5:**
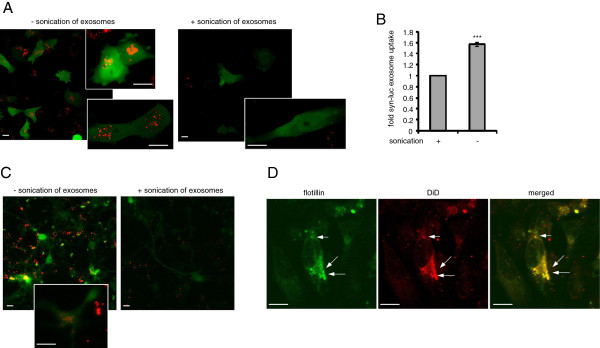
**Exosomes need to be intact to be internalized: (A) DiD labeled exosomes containing αsyn with or without prior sonifications were added to H4 cells transfected with Venus-YFP and incubated for 3 days.** Scale bar = 20 μm (**B**) Exosomal fractions derived from H4 cells transfected with αsyn-luciferase were applied to naive H4 cells after sonication or without sonication and incubated for 3 days. Uptake of αsyn oligomers into recipient neurons was measured performing a luciferase assay on recipient cells. n = 5, one-sample t-test, *p < 0.05. (**C**) DiD labeled exosomes containing αsyn with or without prior sonifications were added to primary cortical neurons infected with AAV-Venus-YFP incubated for 3 days. Scale bar = 20 μm (**D**) Co-localization of exosomal marker flotillin (green) and DiD labeled exosomes (red) after addition of exosomes containing αsyn to naïve H4 cells. Scale bar = 20 μm.

Because our data suggest that exosome-associated αsyn may be preferentially taken up by neighboring cells, we next asked whether exosomes need to be intact for uptake to occur. To explore this question, we labeled purified exosome-enriched fractions derived from S1/S2 transfected H4 cells with the membrane dye DiD [68]. To delineate the morphology of H4 cells or primary neurons, we transfected cells with venus-YFP prior to exosome addition resulting in a subpopulation of H4 cells or primary neurons that could be identified via green fluorescence. As expected when labeled exosomes were exogenously added to H4 cells or primary neurons in culture, we observed a rapid uptake of labeled exosomes into the cytosol of cells. To investigate whether membrane integrity is important for uptake, exosomes were subjected to sonication which is known to disrupt lipid bilayer integrity [69-71]. Sonication of exosomes prevented the uptake of exosomes by recipient cells (Figure 5A). These results were confirmed by measuring luciferase activity inside naive cells following incubation with intact or sonicated exosome fractions from syn-luc transfected H4 cells. Where exosomes were sonicated, significantly less luciferase activity was detected inside the naive cells (Figure 5B) although sonication itself had no effect on luciferase activity of exosomal fractions. This uptake was not unique to proliferating cells as we were also able to confirm the uptake of labeled exosomes into primary cortical neurons (Figure 5C). These results demonstrate that intact exosomes promote exosomal uptake.

To confirm that the labeled microvesicles being observed by confocal microscopy were actually exosomes, we immunostained cells with the exosomal marker flotillin and found that the flotillin immunoreactivity colocalized with the DiD labeled exosomes (Figure 5D).

### Autophagy regulates αsyn secretion

Exosomes are derived from multivesicular bodies (MVBs), which are endocytic organelles generated by membrane invagination [72-74]. Proteins that are designated for lysosomal degradation are sequestered by MVBs, however, an alternative destination of MVBs is their exocytic fusion with the plasma membrane leading to the release of intraluminal vesicles (ILVs; i.e., exosome) into the extracellular environment. Interestingly, induction of autophagy can markedly increase the interaction of MVBs and autophagosomes and thereby block exosome secretion [75].

**Figure 6:**
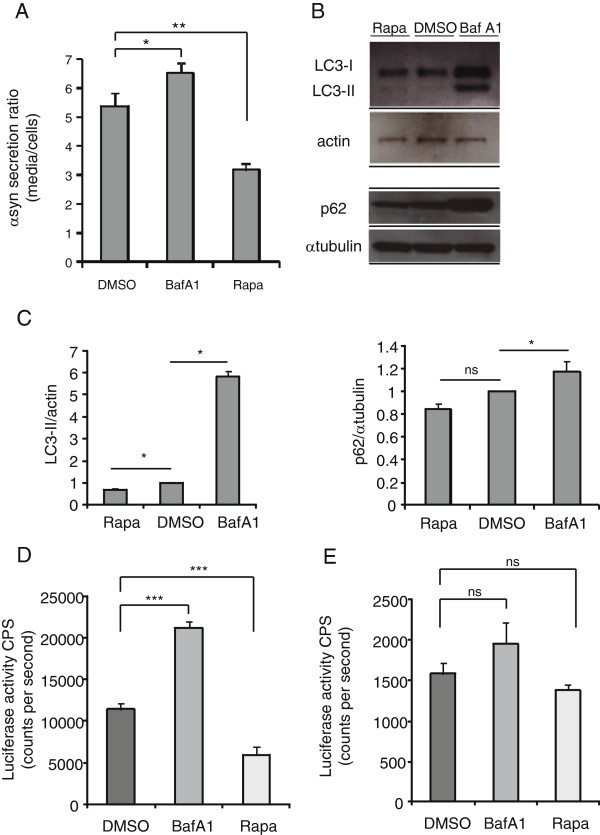
**αSyn oligomer secretion is modulated by autophagic activity. **(**A**) Human H4 cells were transfected with αsyn complementation pair S1/S2 and treated with DMSO, rapamycin or bafilomycin A1. After 48 h cells CM was assayed for luciferase activity and a ratio of luciferase activity in media compared to cells was expressed. n = 4, One way ANOVA, Bonferroni’s Multiple Comparison Test, **p < 0.001. (**B**) Immunoblot levels of autophagosome marker LC3.II and macrophagy substrate p62 in H4 cells treated with autophagy inhibitor Bafilomycin A1, autophagy inducer rapamycin or DMSO. (**C**) Densiometric analysis of immunoblots probed with LC3.II normalized to ß-actin[n = 3 (Baf A1) p < 0.0001, t-test; n = 3 (rapa), p = 0.0021, t-test ] and p62 normalized α-tubulin [n = 3 (Baf A1) p = 0.022, t-test; n = 3 (rapa), p = 0.0022, t-test ](**D**) Exosomal fractions from H4 cells transfected with S1/S2 and treated with DMSO, rapamycin or bafilomycin A1 were assayed for luciferase activity. n = 4, One way ANOVA, Bonferroni’s Multiple Comparison Test, ***p < 0.001 (**E**) Exosomal fractions from primary neurons infected with AAV-S1/S2 and treated with DMSO, rapamycin or bafilomycin A1 were assayed for luciferase activity. n = 4, One way ANOVA, Bonferroni’s Multiple Comparison Test, p = 0.17, ns = non-significant.

We detected a higher luciferase activity extracellularly (Figure 6A) which may suggest that more oligomers are secreted. However, it’s also possible that coelentrazine is more sensitive to extracellular αsyn oligomers although it is cell permeable. Nevertheless, given that we detected αsyn oligomers in the exosomal fraction of cells and observed that αsyn oligomer secretion could be modulated by autophagic activity (Figure 6A) we asked whether autophagy would be a release pathway for exosome-associated αsyn oligomers.

Following guidelines for assays monitoring autophagy [76] we measured the levels of LC3.II (ratio with ß-actin) and p62 (ratio with α-tubulin) in S1/S2 transfected H4 cells treated with rapamycin, bafilomycin A1 and DMSO. Levels of LC3.II and p62 significantly increased when H4 cells were treated with bafilomycin A1 compared to DMSO controls, indicating an abundance of autophagosomes due to reduced downstream fusion with lysosomes. The opposite effect was observed when treated with rapamycin (Figure 6 B, C). We speculated that an increased pool of autophagosomes could be the basis for an increased exosomes secretion. Exosomes were isolated from conditioned media of S1/S2 transfected H4 cells treated with bafilomycin A1, rapamycin or DMSO, and luciferase activity was monitored. A significant increase in luciferase activity in the exosomal fraction from cells that were treated with bafilomycin A1 compared to DMSO control (Figure 6D) was observed which suggests that inhibition of the fusion of the autophagosome with the lysosome by bafilomycin A1 provides an increased pool autophagosomes which enhances exosomal release. Likewise, treatment with the autophagy enhancer rapamycin showed a decreased αsyn oligomer signal in the exosomal fraction as measured by luciferase activity compared with DMSO treatment (Figure 6D), suggesting that enhanced lysosomal activity results in effective αsyn oligomer degradation and less secretion. We extended these findings to primary neurons and observed an increase in the αsyn oligomer signal in the exosomal fraction from neurons treated with bafilomycin A1 and a decrease in the αsyn oligomer signal with rapamycin treatment in the exosomal fraction compared to DMSO control (Figure 6E), although these effects did not reach statistical significance (p = 0.17), probably due to the fact that the yield of exosomes are significantly less from primary neuronal preparations resulting in barely detectable luciferase and an decrease in the signal/noise ratio. Together, these experiments indicate autophagy can be a specific release pathway for secretion of αsyn oligomers.

## Discussion

Multivesicular bodies (MVBs) and their intraluminal vesicles (ILVs) are involved in the sequestration of proteins destined for degradation in lysosomes. However, MVBs can also fuse with the plasma membrane leading to the release of 50-90 nm ILVs into the extracellular milieu, which are then called exosomes [77,78]. Exosome secretion can therefore be used by cells, including neurons and astrocytes, to clear molecules originally destined for lysosomal degradation [77]. Recently, exosomes have been suggested to play a role in neurodegeneration: Exosomes from prion infected cells have been demonstrated to be efficient initiators of prion propagation in uninfected recipient cells and more importantly, to produce prion disease when inoculated into mice [34,79]. Also the beta-amyloid peptide has been found to be secreted from cells in association with exosomes [80]. Ghidoni et al. suggested that exosomes could be the "Trojan horses" of neurodegeneration; a mechanism underlying the death of cells by shipping toxic agents in exosomes from cell to cell [81]. In our study we identified αsyn *oligomers* to be present in exosomes and found that exosome-associated αsyn oligomers are more toxic to neighboring cells than exosome-free αsyn oligomers. In contrast to the study of Hasegawa et al. we found that αsyn oligomers are present in both the exosomal pellet and the exosome free supernatant from the conditioned media of αsyn overexpressing cells whereas Hasegawa et al. recovered αsyn mainly from the supernatant fraction [82]. One possible explanation for this discrepancy is the different cellular models and resulting levels of sensitivity. The use of a highly sensitive luciferase protein complementation assay, allows the detection of minimal amounts of protein compared to Western blot analyses.

**Figure 7:**
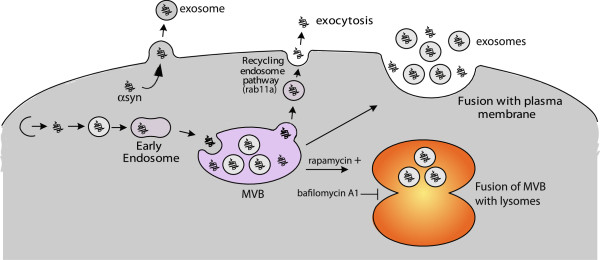
**Schematic presentation of endosomal pathways and possible αsyn secretory pathways.** Membrane associated cargo proteins including αsyn or cytosolic αsyn are translocated to early endosomes. Invagination and final scission of the endosomal membrane contribute to MVB formation. MVB direct either for lysosomal degradation by fusion from the autophagosome with the lysosome or for secretion as exosomes by exocytic fusion with the plasma membrane. Under physiological conditions αsyn in the early endosome may be transferred to MVB and then targeted for lysosomal degradation. Alternatively, αsyn might be secreted into the extracellular milieu through the rab11a dependent recycling endosome (free αsyn) and MVB-exosome pathway (free and exosome associated αsyn). Exosomes can also enter in the extracellular interspace by outward budding of the plasma membrane.

To our knowledge, this is the first report of αsyn *oligomers* in the exosomal fraction of primary neurons or neuronal cells. Specifically, we have conclusively shown that αsyn oligomers can be found outside exosomes, presumably on the outer surface of exosomes. The existence of αsyn oligomers outside exosomes has been demonstrated in two ways. First, any external αsyn has been digested using trypsin which resulted in a significant decrease in luciferase counts indicative for αsyn oligomers. Second, trypsin digestion also led to a dramatic decrease of αsyn signal in a Dot blot approach. There are several possible reasons for the existence of αsyn oligomers on the external surface of exosomes (Figure 7). First, lipid raft components were found on the membrane surface of secreted exosomes [83]. αSyn has been shown to interact with lipid rafts and artificial membranes [84,85] and a recent report demonstrates that αsyn can penetrate in the outer leaflet of a bilayer [86]. 

We also found αsyn present in the inside of exosomes. Cytosolic proteins like αsyn can end up in ILVs because plasma membrane invagination occurs during endocytosis and results in the formation of early endosomes encapsulating a significant amount of cytosol (Figure 7). Endosomal membranes further invaginate to form ILVs, which give endosomes their multi-vesicular appearance. When MVBs fuse with the plasma membrane they can then be released as exosomes containing the encapsulated cytosolic proteins like αsyn [87]. Why αsyn oligomers are encapsulated in MVB’s and released as exosomes is not understood. Possible explanations include first, that αsyn oligomers in exosomes could represent the species that are most harmful to cells and is thus targeted for release. Second, Fang et al. have proposed that proteins which exhibit higher order oligomerization and are associated with the plasma membrane, are preferentially sorted into exosomes [88]. A third explanation for the presence of αsyn oligomers in exosomes could be that exosomes provide an environment that is favorable for the oligomerization process. Indeed, lipid-mediated oligomerization seems to be important in amyloid formation and polyunsaturated fatty acids have been shown to trigger multimerization of recombinant αsyn [89].

We also found αsyn oligomers in the exosome-free fraction. One explanation for the presence of exosome-free αsyn oligomers could be that the the exosomal membrane undergoes extracellular degradation via proteases or lipases that would lead to the release of proteins from the exosomal lumen to the extracellular environment during the fractionation process [90,91]. Another explanation could be that αsyn oligomers might become unstable and lose their affinity for lipid membranes after being released from the cell, due to changes in the pH value or ionic strength in the extracellular space. In fact, only a small fraction of Abeta peptide is found associated with exosomes [80], which supports our observations. A third explanation for the secretion of soluble, non-exosomal αsyn oligomers could be that there is an exosome-independent pathway of αsyn secretion (Figure 7) maybe through the Rab11a-dependent recycling endosomal pathway [82,92], however, further in depth studies will be needed to determine if this is the case. Supporting evidence for the presence of αsyn in the exosomal fraction also comes from the recent studies of Emmanouilidou et al. and Alvarez-Erviti [23,24]. Taken together with the previously published studies, our findings support the "Trojan horse" hypothesis [81].

Our data make a case for exosomal transfer of αsyn from cell to cell and could represent a key mechanism in the spread of αsyn aggregates between neurons in the brain. Indeed, exosomes are biologically active vesicles that are thought to be important for intercellular communication [93,94]. Valadi et al. recently reported that exosomes also contain both mRNA and microRNA, which can be delivered to neighboring cells and be functional in the recipient cell [40]. Exosomes can interact with recipient cells in different ways, including endocytosis, fusion with the plasma membrane, receptor-ligand-binding or attachment [78,95-97].

Critical to our understanding of the toxic effects of secreted αsyn oligomers on neighboring cells is the identification of the toxic species. Our data support the possibility that both exosome-associated αsyn oligomers and exosome-free αsyn oligomers can confer toxicity on neighboring cells. We found that exosome-associated αsyn oligomers are more likely to be taken up by neighboring cells, although we also observe the uptake of free αsyn oligomers. The same holds true in terms of toxicity: exosome-associated αsyn oligomers are more toxic to neighboring cells compared to free αsyn oligomers. It is tempting to speculate that the more αsyn oligomers that are taken up by recipient cells, the greater the toxicity. However, it remains to be determined whether αsyn oligomers exert their toxic effects after being taken up by recipient cells by impacting cellular homeostasis [98,99] or if extracellular αsyn oligomers exert their effect at the cell membrane.

Autophagy can function as a protective mechanism in cells and is particularly crucial in the aging brain and in neurodegeneration where aggregated proteins accumulate [100]. It is now thought that αsyn can be degraded by either the proteasome or by autophagy and both macroautophagy and CMA have been reported to contribute to αsyn degradation [101-104]. In this study, we demonstrate that secretion of αsyn oligomers is increased when lysosomal activity is blocked by Baf A1. Baf A1 inhibits the fusion of the autophagosome with the lysosome by inhibiting vacuolar type H(+)-ATPase [105], thereby inhibiting lysosomal activity. We speculate that by blocking the major degradation pathway for αsyn oligomers, the cells use secretion as an alternative pathway to eliminate harmful αsyn oligomeric species. By contrast, we did not detect a significant effect of proteasomal inhibition with MG132 on the secretion of αsyn oligomers. These results support a hypothesis where autophagy is the major route for degradation of αsyn oligomers which are then targeted to the plasma membrane to be cleared by secretion as an alternative route upon failure of this pathway. This assumption is also supported by the fact that rapamycin decreased αsyn secretion by enhancing autophagy and thereby triggering intracellular degradation of αsyn oligomers. Our results are also in line with the recent work from Emmanouilidiou et al., who did not observe an effect of proteasome inhibitor on levels of extracellular αsyn, but found a profound increase in the levels of secreted αsyn when the lysosomal pathway was blocked by methylamine [23]. Our study specifically investigates the regulation of secretion of oligomeric αsyn upon autophagy inhibition or activation, supporting and significantly augmenting the published study. The fact that we observed more αsyn oligomers in the exosomal fraction after inhibition with BafA1 raises the possibility that αsyn oligomer containing vesicles (presumably ILVs) originally destined for lysosomal degradation, were re-directed to the plasma membrane and released as exosomes. This hypothesis requires an interaction between exosomal and autophagic pathways. Indeed, a recent study by Fader et al. demonstrated that induction of autophagy markedly increased the interaction of MVBs and autophagosomes and concurrently blocked exosome secretion, suggesting that MVBs are directed to the autophagic pathway with a consequent inhibition in exosome release [75].

In conclusion, we demonstrate that αsyn oligomers can be found in different extracellular fractions in association with exosomes or as exosome-free oligomers. αSyn oligomers associated with exosomes are more toxic to recipient cells compared to free αsyn oligomers. The toxic mechanisms of αsyn oligomers spreading from cell to cell described here in cell culture could resemble events explaining the spread of αsyn pathology that has been observed in human post-mortem brains [8]. Additional studies are needed to verify exosome-associated αsyn oligomers and exosomal release in the brains of PD patients. Preventing the early events in exosomal release and uptake by inducing autophagy might be a novel approach for the development of effective drugs for the treatment of PD and other synucleinopathies.

## Conclusions

These data demonstrate that oligomeric forms of αsyn can be found in multiple extracellular fractions: associated with exosomes and free. Exosome-associated αsyn oligomers are more likely to be taken up by recipient cells and can induce more toxicity compared to free αsyn oligomers. In addition, we determined αsyn oligomers oligomers to be present both on the outside of exosomes as well as inside of exosomes. Notably, the pathway of secretion of αsyn oligomers is strongly influenced by autophagic activity. Preventing the early events in αsyn exosomal release and uptake by inducing autophagy may be a novel approach to halt disease spreading in PD and other synucleinopathies.

## Methods

### Plasmid generation

Fusion constructs αsyn-hGLuc1 (S1), αsyn-hGLuc2 (S2) and Venus1-αsyn (V1S), αsyn-Venus2 (SV2) were generated by subcloning αsyn into Not1/Cla1 sites of humanized Gaussia Luciferase and VenusYFP constructs provided by Dr. Stephen Michnick of University of Montreal [25,28].

### AAV vectors construction and production

The viral vectors rAAV-CBA-WPRE, rAAV-CBA-IRES-EGFP and rAAV-CBA-SYNUCLEIN-IRES-EGFP were described previously [29]. rAAV-CBA-SYNUCLEIN-LUC1-WPRE (AAV-S1) and rAAV-CBA-SYNUCLEIN-LUC2-WPRE (AAV-S2) were constructed as follows: αsyn -hGLuc1 (S1) and αsyn -hGLuc2 (S2) were subcloned into Not1/Nhe1 sites of AAV-CBA-WPRE vector. rAAV-CBA- VENUS1-SYNUCLEIN-WPRE (AAV-V1S) and rAAV-CBA-SYNUCLEIN-VENUS2-WPRE (AAV-SV2) were constructed as follows: the fragments Venus1-Synuclein and Synuclein-Venus2 was inserted into the EcoRV and NheI sites of the pAAV-CBA-WPRE vector. Recombinant adeno-associated type 2/8 was generated by tripartite transfection (AAV-rep/cap expression plasmid, adenovirus miniplasmid and AAV vector plasmid) into 293A cells and purified by iodixanol gradient followed by Q sepharose column chromatography (Harvard Gene Therapy Initiative, Harvard Medical School). The purified virus was dialyzed against PBS, concentrated by Amicon spin column, and tittered by dot blot hybridization. Final titers for virus were for AAV-S1 1.5E13 gc/ml, AAV-S2 1.3E13 gc/ml, V1S 8.3E12 gc/ml and SV2 8.7E12 gc/ml.

### Human αsynuclein ELISA

Alpha synuclein concentration was quantified using human αsyn specific ELISA (#KHB0061, Invitrogen, Carlsbad, CA, USA) according to the manufacturer’s instructions. Absorbance is read at 450 nm. The absorbance is directly proportional to the concentration of αsyn present in the original specimen. αSyn concentration was determined by plotting sample absorbances against standards using Graph Pad Prism fitting software (four parameter algorithm).

### Cell culture and transfections

Unless otherwise stated, human H4 neuroglioma cells (HTB-148 - ATCC, Manassas, VA, USA) were maintained in OPTI-MEM medium supplemented with 10% fetal bovine serum (both from Invitrogen) and incubated at 37°C. Cells were plated 24 hours prior to transfection, growing to 80–90% confluency prior to transfection. Transfection was performed using Superfect (Qiagen, Chatsworth, CA, USA) using equimolar ratios of plasmids according to the manufacturer’s instructions. Conditioned media was collected 48 hours post-transfection and centrifuged for 5 min at 3000 g to eliminate floating cells before being used.

### Gaussia luciferase protein-fragment complementation assay

Fusion constructs αsyn hGLuc1 (S1) and αsyn -hGLuc2 (S2) were generated as described previously [25]. S1 and S2 were transfected into H4 cells in a 96-well plate format as described above. 48 h after transfection, culture media was transferred to a new 96 well plate (Costar, Corning, NY, USA). Cells were washed with PBS and replaced with serum- and phenol-red free media. Luciferase activity from protein complementation was measured for conditioned media and live cells in an automated plate reader at 480 nm following the injection of the cell permeable substrate, coelenterazine (20 μM) (Prolume Ltd, Pinetop, AZ) with a signal integration time of 2 seconds.

### Primary cortical cell culture

Primary cortical neurons were prepared from cerebral cortices of E14-16 mouse embryos. Cortices were dissected from embryonic brain and the meninges were removed. Cortices were dissociated by titruation at RT and cells were resuspended in Neurobasal (NB) (Gibco) medium supplemented with 10% fetal bovine serum, 2 mM Glutamax, 100 U/mL penicillin, and 100 μg/mL streptomycin and plated at a density of 3.84 x 10^4^ cells/well on 96w plates (Corning, NY, USA), 60 mm dishes at a density of 3.6 x 10^6^ cells/dish (Costar, Corning, NY, USA) coated with 20μg/mL poly-D-lysine(Sigma-Aldrich, St. Louis, MO, USA). After 2 h medium was changed into NB/B-27 [NB medium containing 2% (v/v) B-27 supplement], 100 U/mL penicillin, 100 μg/mL streptomycin, and 2 mM/L glutamine. Cells were maintained at 37°C in 5% CO_2_ in a humidified incubator. Medium was changed every third day. Neurons were grown for 4–5 days in vitro (DIV) before infected with AAV8-S1/AAV8-S2, AAV8-V1S/AAV8-SV2, AAV8-syn-ires-GFP or AAV8-GFP. Infections were carried out as followed: 6 μl rAAV2/8 expressing eGFP (1.3E13gc/ml) per 60 mm dish, 6μl rAAV2/8 expressing αsyn(wt)-ires-GFP (1.1E13gc/ml) per 60 mm dish and 3 μl rAAV2/8 expressing V1S (8.3E12gc/ml) together with 3 μl rAAV2/8 expressing SV2 (8.7E12gc/ml) per 60 mm dish, as well as 3 μl rAAV2/8 expressing S1 (1.5E13gc/ml) together with 3 μl rAAV2/8 expressing S2 (1.3E13gc/ml) per 60 mm dish.

### Exosome isolation

Exosomes from Human H4 cells and primary neurons were prepared as described earlier [34,35] with minor modifications. Briefly, conditioned medium was collected and spun for 5 min at 500xg to remove floating cells. The supernatants were then sequentially centrifuged at 300xg (10 min) and 2x 200xg (10 min) at 4°C each. Then supernatants were filtered through a 0.45 μm (Whatmann, Florham Park, NJ) and then 0.22 μm (Millipore, Carrigtowhill, Cork, Ireland) filter, and centrifuged for 30 minutes at 10,000xg (2X) at 4°C. After ultracentrifugation at 100,000xg for 70 min at 4°C, exosomal pellet was then resuspended in 1xPBS for Western Blotting, electron microscopy or luciferase assay or resuspended culture medium for cell treatments.

Exosome depleted medium was prepared as described above, except after ultracentrifigation at 100,000xg for 70 min at 4°C, exosome free supernatant was filtered through a 0.22 μm filter before used in cell culture.

### Digestion of exosomes

Exosome associated or exosome free αsyn oligomers were digested by addition of 0.25% trypsin (Invitrogen, Carlsbad, CA, USA) and/or 0.1% saponin and incubated for 20 min at 37°C. After complete digestion samples were analyzed in luciferase assay or Dot blot approach.

### Labeling of exosomes

The exosomes were labelled using DiD (Biotium, Hayward, CA USA) according to manufacturer’s instructions in a 1:200 dilution. Briefly, after the final spin in exosome purification exosomal pellet was resuspended in 1 ml DiD solution and incubated for 5 minutes. After ultracentrifugation at 100,000xg for 70 min at 4°C the exosomal pellet was washed in 1xPBS centrifuged again for 90 min at 150 000 g to remove free dye. Then the pellet is resuspended finally as exosome fraction.

### Dot blot

Exosomes or exosome free supernatant was collected as described previously. 100ul of each condition was applied to nitrocellulose membrane (pore size 0.22 um, Whatman Protran,Sanford, ME, USA) placed in a Dot blot apparatus (Schleicher & Schuell Minifold-I Dot-Blot System, Whatman, Sanford, ME, USA, ) and incubated at RT for 1 h. Samples were filtered through the membrane by gentle vacuum and developed using conditions as described previously (21). Briefly, the membrane was blocked with 10% non-fat dried milk in Tris-buffered saline (TBS, Sigma-Aldrich, St. Louis, MO, USA) containing 0.01% Tween 20 (TBS-T), at room temperature for 1 h. After three washes with TBS-T, the membrane was incubated with anti- Syn-1 antibody (1:1000; BD transduction, Franklin Lakes, NJ, USA) or monoclonal anti-CD63 antibody (1:500, BD Transduction) overnight at 4 C with gentle agitation. The membranes were then washed three times for 5 min with TBS-T, incubated with horseradish peroxidase conjugated anti mouse IgG (Jackson Immuno Research Laboratories, Baltimore, PA, USA) diluted 1:2000 in 5% non-fat dried milk in Tris-buffered saline (TBS) containing 0.01% Tween 20 (TBS-T) and incubated for 1 hour at room temperature. The blots were washed three times with TBS-T and developed with Pierce ECL chemiluminescence kit from Thermo Scientific (Rockford, IL, USA).

### Pharmacological treatments in vitro

H4 cells were plated into 96 well plates or 60 mm dishes (Costar, Corning, NY, USA) and transfected as described above. Transfection mix was incubated for 2 h according to manufacturer’s protocol, then media was replaced by fresh culture media containing 0.4 μg/ml rapamycin (Sigma Aldrich, St. Louis, MO) or DMSO (Sigma Aldrich, St. Louis, MO) and incubated for 48 h. 200nM Bafilomycin A1 (Merck KG, Darmstadt, Germany) was added to the culture medium 20 h before harvesting the medium. Conditioned medium was collected for aluciferase assay or exosomal isolations. To ensure that pharmacological treatments result in a true increase in the secretion ratio of αsyn oligomers and not simply a matter of more available αsyn oligomers in the cytoplasm, we calculated the ratio of secreted αsyn oligomers in the conditioned medium to intracellular αsyn oligomers.

Primary neurons were plated into 60 mm dishes (Costar, Corning, NY, USA) and infected as described above. DMSO and 0.1 μg/ml rapamycin were added to the culture medium right after infection, whereas 100nM bafilomycin A1 was added after 3 days expression and incubated for 20 h. Conditioned medium was then collected to perform exosomal isolations.

### Exosomal uptake experiments

Conditioned media from naïve H4 cells or naïve primary neurons was replaced by exosome containing culture media. After 3–4 days incubation cells were washed twice with 1xPBS and then assayed for luciferase activity.

### Toxicity assay

Toxicity was analyzed 3–4 days after exosome application by measuring the activity of Caspases 3 and 7 using a fluorometric substrate Z-DEVD-Rhodamine 110 (Apo-ONE homogeneous Caspase-3/7 assay #G7790, Promega, Madison, WI) according to the manufacturer’s protocol.

### Western blotting

Primary cortical neurons were scraped from 60 mm dishes and washed by centrifugation and resuspension in cold PBS. The cells were resuspended in 1x PBS containing protease inhibitors (protease inhibitor cocktail tablet 1 tablet/10 mL (Roche Diagnostics) sheared by passing through a 27-gauge 1 ml syringe 4–6 times and centrifuged for 5 min at 13,000 g. Lysates or exosomal samples were resolved by electrophoresis on a 4–12 % Bis-Tris gradient gel (NuPAGE Novex Bis-Tris Gel, Invitrogen, Carlsbad, CA, USA) according to manufacturer’s instructions using NuPAGE MOPS buffer. After transfer to nitrocellulose membrane (Protran, Schleicher and Schuell, Whatman GmbH, Dassel, Germany) membranes were blocked in either 5% milk inTBS-T or Li-Cor blocking buffer (LI-COR,Lincoln, NE, USA) for 1 hour at room temperature. Membranes were then incubated with primary antibodies (mouse anti-Alix, 1:500, BD Transduction; mouse anti-flotillin: monoclonal, 1:500, BD Transduction) overnight at 4 C. After three 5–10 min TBS-T washes, membranes were incubated at room temperature for 1 hour with either IR-labeled secondary antibodies (IR800 goat anti-mouse, 1:2000, Rockland Immunochemicals, PA,USA) or HRP-conjugated secondary antibodies (1:2000). After three 5– 10 min TBS-T washes, immunoblots were analyzed using either the Odyssey Infrared imaging system (Li-Cor, Lincoln, NE,USA) or the ECL chemiluminescent detection system (Amhersham/GE HealthCare, Buckinghamshire, UK).

### Cell imaging and immunofluorescence staining

All images were acquired using a 20x Plan Apochromat lens (Carl Zeiss), 25x APO-Plan NEOFLU lens (Carl Zeiss) or Zeiss 63x 1.2 NA C-APO-Plan NEOFLU water immersion lens (Carl Zeiss), mounted on the microscope described before. H4 cells or cortical neurons were washed three times with phosphate-buffered saline (PBS) following 30 min incubation in a fixation solution containing 4% paraformaldehyde in PBS. After washing, the cells were permeabilized and unspecific binding sites were blocked using 0.05% Saponin and 1% bovine serum albumin in PBS followed by another washing step. The primary rabbit antibody against flotillin (1:500, BD Transduction) was added for 1 h at RT, followed by another washing step and incubation with the secondary antibody (anti-mouse antibody labeled with Alexa-Fluor 488; Invitrogen) for 1 h at RT.

### Electron microscopy

An exosome pellet from either human H4 cells or primary neurons was prepared by centrifugation as described above and then resuspended in 20 μl of cold Karnovsky’s EM fixative (2% formaldehyde and 2.5% glutaraldehyde in 0.1 M Sodium Cacodylate buffer, pH 7.4.). Ultrathin sections from LR white embedded samples were picked up from the knife with a loop, dipped in a 2:1 mixture of 2.3 M sucrose and 2% methylcellulose, and adsorbed to the surface of a formvar/carbon coated copper grid. Grids are placed on 2% gelatin in a small petri dish and stored in the fridge until immunogold labeling. This was accomplished by washing grids in PBS and then either treating with CD63 antibody (BD Transduction) followed by 10 nm gold labelled secondary antibodies (Sigma Aldrich, St. Louis, MO) or processing without immunolabelling. These exosome containing grids were then post-fixed with 2.5% glutaraldehyde, washed and contrasted with 2% methyl cellulose and 3% aqueous uranyl acetate. Samples were examined and photographed with a JEOL 1200EX electron microscope.

### microRNA profiling and data analysis

For nucleic acid analysis, the entire exosome pellet is gently resuspended in 20 μl of 1xPBS. Any cellular/ribosomal RNAs that may exist in the extra-exosomal solution (53) are eliminated by adding 8 μg of RNAse T1/A (Fermentas) to the 20ul preparation and incubating for 10 minutes at 37°C. Four hundred units of SuperRase-in RNAse (Ambion) inhibitor are then added to inactivate the RNAses and the sample is held at 25°C for 10 minutes. The entire mixture is then dissolved in 60 μl of miRNA extraction buffer (Arcturus), incubated at 42°C for 30 minutes, and stored at −80°C prior further processing. In order to generate amplified sense RNA from small quantities (<1 ng) of purified miRNAs, we used the NCode miRNA Amplification System (Invitrogen) according to the manufacturer’s instructions. miRNA expression profiles were generated by adding 250 ng of this amplified miRNA to the FlexMiR miRNA assay from Luminex Corporation (Austin, Texas) and running on a Luminex FlexMAP 3D system according to the manufacturer's instructions.

### Statistical analysis

Statistical analyses were carried out using the program GraphPad Prism, Version 4.0. Values in the figures are expressed as means +/− SEM.

## Abbreviations

PD: Parkinson’s disease; αsyn: Alpha synuclein; CM: Conditioned media; V1S: Venus YFP fused to the N-terminus of αsyn; SV2: C-terminal half of Venus YFP fused to the C-terminus of αsyn; S1: αsyn fused to amino-terminal fragment of *Gaussia princeps* luciferase; S2: αsyn fused to carboxy-terminal fragment of *Gaussia princeps* luciferase; MVBs: Multivesicular bodies; CSF: Cerebrospinal http://fluid.

## Competing interests

The author declares that they have no competing interests.

## Authors’ contributions

KMD, LRK, WPR, OCG, ARW, LZ and CRV performed the experiments. KMD and PJM and analyzed the results, designed the study, and wrote the manuscript. All authors read and approved the final manuscript.

## Supplementary Material

Additional file 1 **Schematic representation of the αsyn protein fragment complementation constructs.** (A) Nonbioluminescent halves of humanized gaussia luciferase are fused to αsyn monomers (B) Non fluorescent halves of Venus-YFP are fused to αsyn monomers. Click here for file
