# Supplementary figures and images for: Exosomal cell-to-cell transmission of alpha synuclein oligomers
Source: Mol Neurodegener. 2012 Aug 24;7:42. doi: 10.1186/1750-1326-7-42 (PMC3483256; doi:10.1186/1750-1326-7-42)

**A**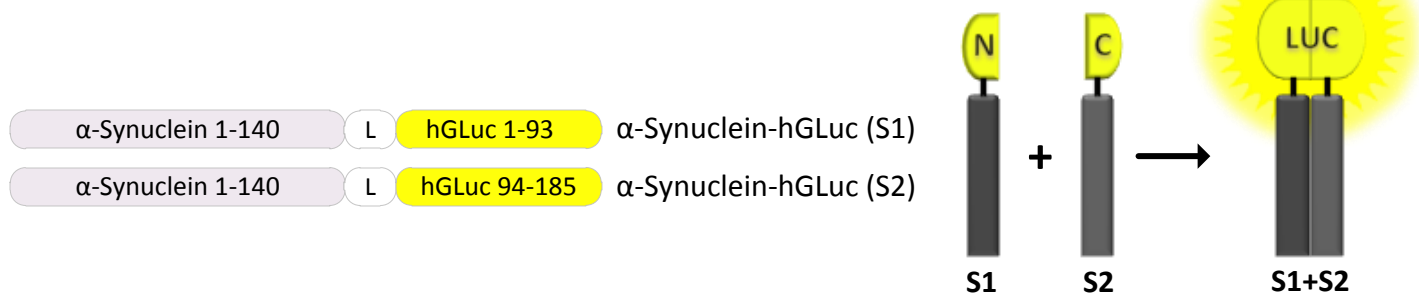**B**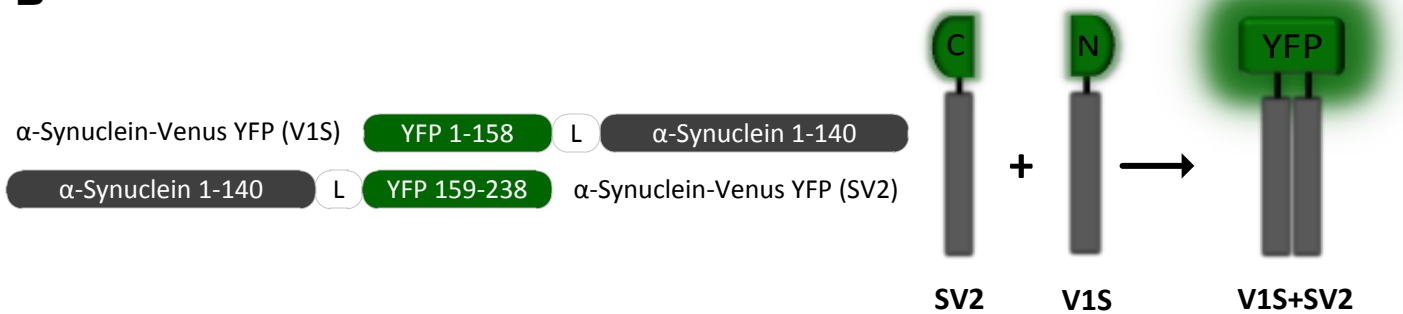

Supplement: Additional file 1 — Schematic representation of the αsyn protein fragment complementation constructs. (A) Nonbioluminescent halves of humanized gaussia luciferase are fused to αsyn monomers (B) Non fluorescent halves of Venus-YFP are fused to αsyn monomers. [file 1750-1326-7-42-S1.pdf]
